# Distal radius fractures—Regional variation in treatment regimens

**DOI:** 10.1371/journal.pone.0207702

**Published:** 2018-11-16

**Authors:** Jenny Saving, Sari Ponzer, Anders Enocson, Cecilia Mellstrand Navarro

**Affiliations:** 1 Department of Clinical Science and Education, Södersjukhuset, Karolinska Institutet, Stockholm, Sweden; 2 Department of Orthopaedic Surgery, Södersjukhuset Hospital, Stockholm, Sweden; 3 Department of Molecular Medicine and Surgery, Karolinska University Hospital, Stockholm, Sweden; 4 Department of Hand Surgery Södersjukhuset Hospital, Stockholm, Sweden; Medical College of Wisconsin, UNITED STATES

## Abstract

**Objectives:**

After recent technical innovations of fracture surgery implants, treatment traditions are changing for distal radius fractures, the most common orthopaedic injury. The aim of this study was to determine if the choice of surgical method for treatment of distal radius fractures differ between healthcare regions in Sweden.

**Method:**

The study was based on all (n = 22 378) adult patients who were registered with a surgical procedure due to a distal radius fracture during 2010–2013 in Sweden. Consecutive data was collected from the Swedish National Patient Registry.

**Results:**

The proportions of use of surgical method varied among the 21 healthcare regions between 41% and 95% for internal fixation, between 2.3% and 44% for percutaneous fixation and between 0.6% and 19% for external fixation. Differences between regions were statistically significant in all but 6 comparisons when controlled for age and gender. Incidence rates of surgical treatment of a distal radius fracture varied between 4.2 and 9.2/10 000 person-years.

**Conclusion:**

We conclude that there is a large variation in operative management of distal radius fractures between Swedish healthcare regions.

## Introduction

For a distal radial fracture, the most common of all fractures, there is no consensus regarding optimal treatment [1]. Benign non-displaced fractures are commonly treated non-operatively in a plaster cast. Unstable fractures tend to heal in a non-favorable anatomical position, and surgical treatment is usually recommended [2]. In Sweden, 20–30% of radius fractures are treated surgically [3, 4]. Different surgical methods are available, all with their inherent advantages and disadvantages. Percutaneous pinning involves closed reduction of the fracture, and metal pins are inserted through the skin into the fractured bone to reduce and fix the fracture fragments. Cast and pins are removed after 5–6 weeks. External fixation (EF) constitutes a scaffold of metal kept outside the skin for 6 weeks during fracture healing. It is held in place by metal rods in the radial diaphysis and second metacarpal bone and establishes a traction over the wrist, aligning the fractured bones. EF can be augmented with percutaneous pins to add stability or to reduce intra-articular fractures. Commonly reported complications after EF and/or pinning are superficial nerve palsy, pin tract infection and loss of fracture reduction [5, 6]. However, severe complications are rare [7]. At the turn of the century the innovation of plates with locking screws provided new surgical possibilities for fracture fixation, even in comminuted fractures and osteoporotic bone. The skin is opened at the volar side of the wrist, the median nerve and the radial artery are retracted, and the fracture is exposed, reduced and fixed by a plate held in place by screws. Volar plating allows early motion of the wrist which seems to be beneficial for successful postoperative rehabilitation. However, the complication rate has been reported to be high including median nerve palsy, tendon irritation and tendon rupture [8, 9].

Since the introduction of the plates with locking screws, the incidence rate of surgically treated distal radius fractures has increased in Sweden [3, 10] and other countries [11–13]. There are numerous publications showing advantages with plating as compared to traditional percutaneous methods for fracture fixation [14–20], but the magnitude of these differences do not reach a minimal clinically importance [21–25]. Nevertheless, reports from the US and Scandinavia reveal an increase in the use of plating as a surgical treatment of a distal radius fracture [3, 10, 12, 26]. Local traditions and personal preferences on behalf of the surgeon largely affect the choice of treatment [26, 27]. Large regional variation has been shown to exist in the treatment regimens for distal radius fractures in the US [13, 28, 29] and in the Netherlands [30].

The aim of this study was to investigate whether the choice of surgical methods for distal radius fractures in Sweden varied between different healthcare regions.

## Materials and methods

This is a nation-wide registry study investigating all patients that were registered with a surgical treatment due to a distal radius fracture in Sweden during the years 2010–2013. Patients and treatments were identified in the Swedish national patient register (NPR), kept by the Swedish National Board of Health and Welfare (www.socialstyrelsen.se). Swedish healthcare providers mandatorily report all surgical interventions performed in Sweden to the NPR since 1990 [31]. An accuracy of the data in the NPR has been approximated to 90% by validating studies [31].

**Fig 1:**
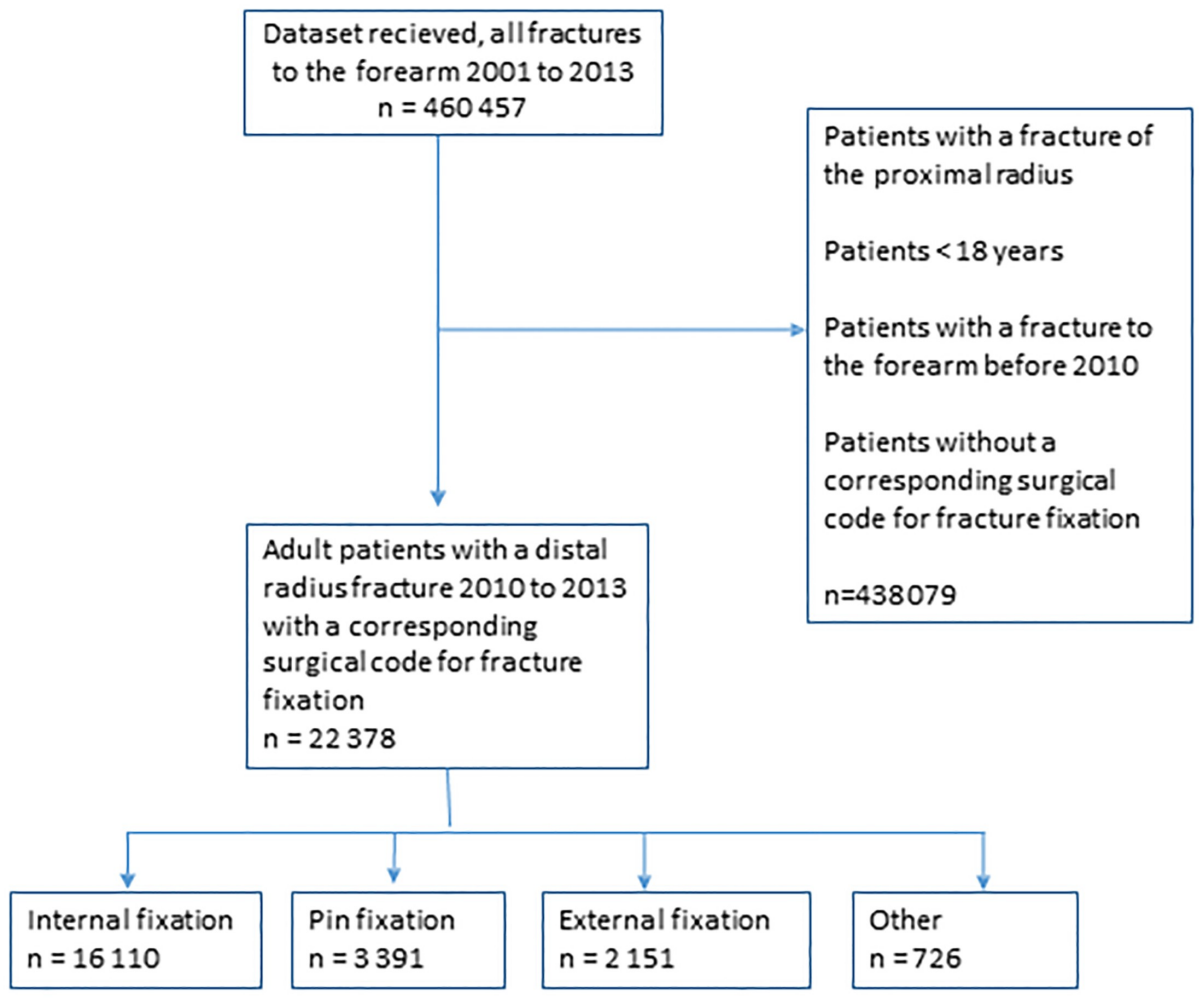
Flow chart with the inclusion and exclusion criteria for the study population; adult patients in Sweden with a surgically treated distal radius fracture 2010–2013.

All registry data regarding individuals with a diagnosis ‘Forearm fracture, S52 (International Statistical Classification of Diseases and Related Health Problems—Tenth revision, ICD-10)’ was identified in the NPR during the period 2001–2013. We defined the study period under investigation to 2010–2013. Each individual patient was recognized by the unique personal identity number used by all Swedish citizens in every contact with healthcare. The geographical location of where each fracture and its treatment took place was defined by the unique code that each of the 21 politically and economically independent healthcare regions in Sweden uses when reporting patients to the NPR register. Only patients 18 years of age or older were included. Records with a diagnosis ‘Distal radius fracture’, S52.5 or S52.6, and a concurrent code for surgical fracture treatment of the hand or forearm were selected. Registry variables retrieved and analyzed included age, sex, type of surgical intervention according to the Nordic codes for surgical procedures (NOMESCO), and date of admission. We limited our analysis to the first occasion when a patient was recorded with a surgical treatment for a distal radius fracture, in order to avoid inclusion of reoperations or new distal radius fractures of the same or the contralateral side as the choice of surgical method might be influenced by the previous method. A flowchart for patient selection is presented in Fig 1.

Four groups of surgical treatments were defined: internal fixation (volar, radial, dorsal or combined plating) with or without pins (IF), pin fixation only (PF), external fixation with or without pins (EF), and other treatments or combinations of methods (Other). The total number and proportion (number in each surgical treatment group/total number of operations) for each surgical method were calculated for each region 2010–2013.

### Statistics

IBM SPSS Statistics version 23 was used for handling of data and calculations of proportions. A multinomial logistic regression was performed controlling for age over 60 and gender, choosing plate fixation in Örebro region as the reference. Significance was considered as p <0.05. Incidence rates were calculated as the number of surgeries divided by the population in each region on 1 November each year according to Statistics Sweden (SCB) (www.scb.se).

### Ethics

The study was approved by the local Ethics Committee of Stockholm, Sweden (reference numbers 2014/1044-32, and 2010/1760-31/4).

## Results

### The total population

22 378 individuals were identified with a surgically treated distal radial fracture in Sweden during the period 1 January 2010–31 December 2013 (Fig 1).

The total distribution of surgical techniques used are presented in Table 1.

**Table 1:** Distribution of surgical procedures for distal radius fractures in Sweden 2010–2013.

	Internal Fixation	Percutaneous Fixation	External Fixation	Other	Total
**Number (%)**	16110 (72)	3391 (15)	2151 (10)	726 (3)	22378 (100)

The mean incidence rate of surgical treatment of a distal radial fracture in Sweden during the period 2010–2013 was 7.4/10 000 person-years (Table 2).

**Table 2:** Incidence of surgical treatment of a distal radius fracture per 10 000 person-years in different regions in Sweden during 2010–2013.

Region	Incidence per 10 000 person-years
Blekinge	4.2
Värmland	4.6
Skåne	5.5
Kronoberg	5.6
Östergötland	5.6
Gävleborg	6.5
Västmanland	6.5
Halland	6.6
Jönköping	7.5
Uppsala	7.7
Dalarna	7.8
Götaland	7.8
Stockholm	8.3
Västerbotten	8.4
Västernorrland	8.5
Jämtland	8.6
Gotland	8.7
Sörmland	8.7
Kalmar	8.9
Örebro	9.1
Norrbotten	9.2
**Total**	**7.4**

### Regional populations

Incidence rates of surgical treatment of a distal radius fracture varied among the 21 healthcare regions in Sweden between 4.2 and 9.2/10 000 person-years. (Table 2).

**Fig 2:**
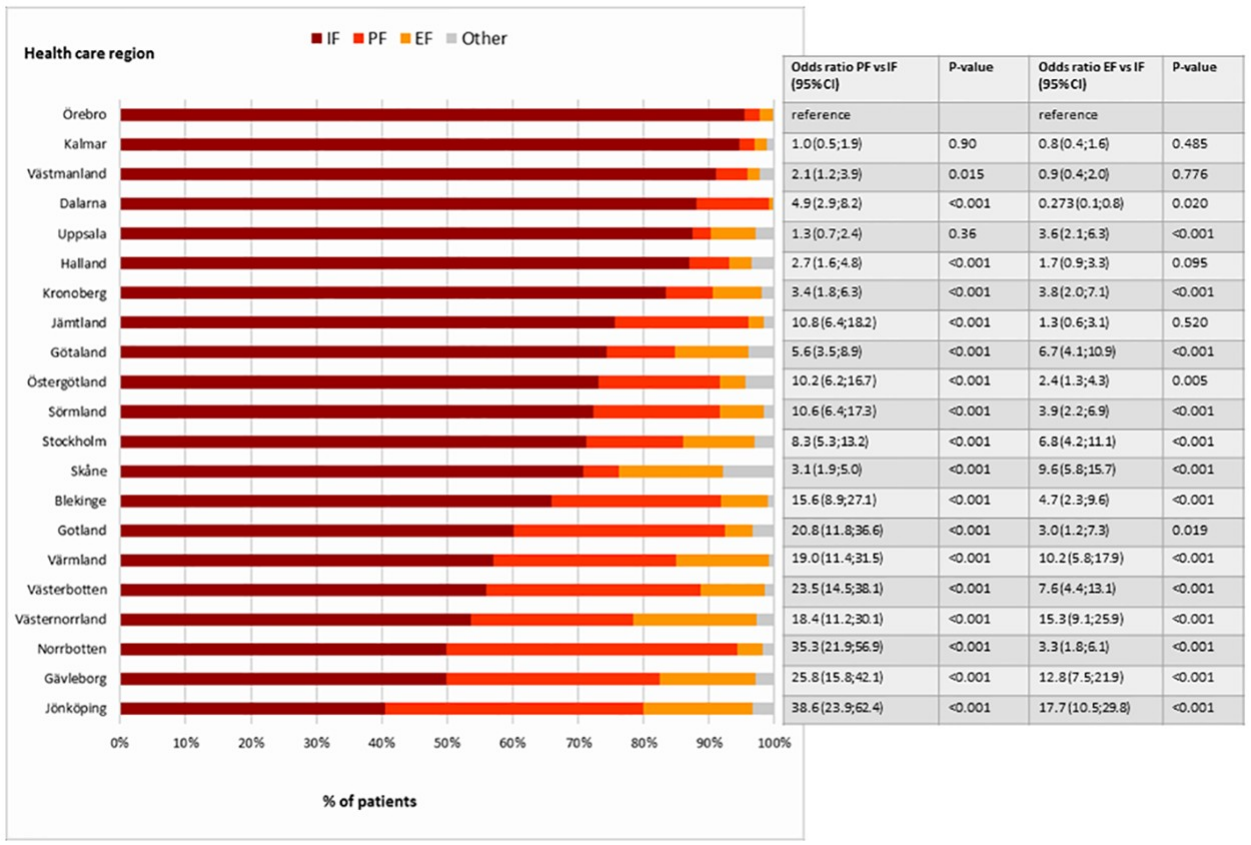
Proportions of patients treated surgically for distal radius fractures with different methods. Patients are adults in each of the 21 health care regions in Sweden during 2010–2013 (IF internal fixation, PF pin fixation, EF external fixation). Multinomial logistic regression performed with the proportion of surgical management with internal fixation in Örebro region (the highest proportion) as the reference.

The proportion of use of IF ranged from 41% (Jönköping) to 95% (Örebro). The proportion of PF ranged from 2.3% (Örebro) to 44% (Norrbotten). The proportion of EF ranged from 0.6% (Dalarna) to 19% (Västernorrland) (Fig 2). Differences were significant in all but 6 comparisons when controlled for age and gender.

**Fig 3:**
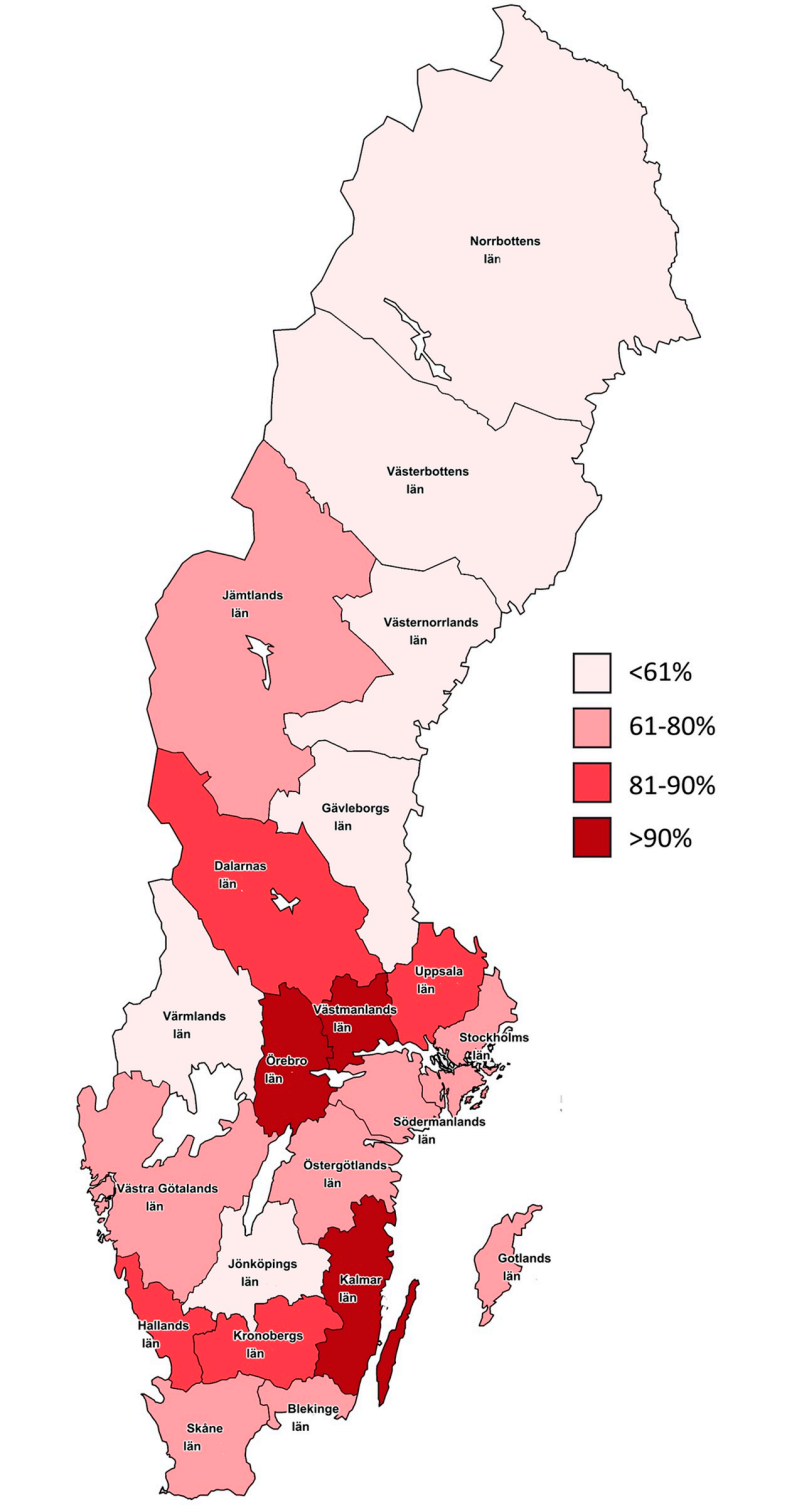
Proportion of internal fixation in surgically treated distal radius fractures in adults for each healthcare region in Sweden during 2010–2013.

The highest IF frequencies were reported from regions situated in the south of Sweden. The regions reporting the lowest IF frequencies were all except one situated in the northern parts of Sweden (Fig 3).

## Discussion

The major finding in this study was that there were large regional differences in surgical treatment preferences for distal radius fracture care between different health care regions in Sweden during the period 2010-2013. The differences remained significant after adjustment for sex and age.

The reasons for the treatment differences between regions found in our study have some possible explanations. Since the current study was conducted with registry data without information on fracture classification we cannot rule out the possibility that different regions had different case mixes, with more or less complicated fractures in different regions. This, however, does not seem likely. Another explanation may be that personal preferences among surgeons, and local treatment traditions dictate most of the treatment decisions. There is support in the literature that such circumstances exist [26, 27, 32]. In the case of scarce scientific evidence of optimal treatment this may be an acceptable and reasonable ground for decision-making. In the case of distal radius fractures, it is somewhat surprising that large multicenter studies and/or meta-analyses have failed to discern optimal treatment algorithms for distal radial fracture care, which would minimize regional variation in treatment choices. An optimal scenario for large patient populations treated in any health care system would be that treatment recommendations were created based on solid scientific evidence regarding treatment effects and risks and cost effectiveness studies respectively, with a possibility of influence from patients’ personal preferences in the decision-making. In the case of distal radius fracture, there is a lack of national consensus regarding treatment recommendations, which might be reflected by our findings of large regional differences in treatment traditions. There is a need for more well-designed controlled comparative studies regarding distal radius fractures to create a solid scientific ground for future treatment guidelines.

There are studies from other countries showing similar regional differences in treatment traditions for radius fractures as in our study. Walenkamp et al [30] report that the choice of non-operative or operative treatment in different regions in the Netherlands differed largely, and the differences were not found to be explained by other factors than personal preferences of the surgeons. In accordance with Walenkamp’s findings, Chung et al [29] reported large differences between regions in the US in treatment choices for radius fractures, not explainable by age, sex or fracture type. In the same study, Chung reported that in the US in 2007, 66% of all surgically treated distal radius fracture patients were treated with internal fixation, and 29% and 5% with pins or external fixation respectively. This is in line with our findings in Sweden 2010–2013 showing an approximately equal distribution of total proportions of surgical methods between these two countries.

Neuhaus et al [33] present a world wide web-based survey interviewing orthopedic and hand surgeons regarding indication for surgery in wrist fractures. They found that surgeons chose non-operative treatment more often in Europe and North-America than in Asia and Australia showing that there are differences of treatment algorithms for distal radius fractures not only within countries, but also between countries and continents.

Chung et al [34] concluded in their study that the degree of education matters when decision-making is performed regarding indication for surgery of radial fractures. Patients treated by American Society for Surgeons of the Hand (ASSH) members received internal fixation significantly more often than patients who were treated by surgeons who were not ASSH members, when controlled for other influencing factors such as fracture type, patient age or gender. There is support in other studies [32, 35] that physician education matters for definition of indications for surgical treatment.

We present data suggesting that indications for surgical treatment differ between regions, and this finding is supported by other authors [30]. In our study there was a two-fold difference in incidence of surgical treatment of distal radius fractures between some regions. The regions with a high incidence of surgical treatment treat a larger proportion of fractures surgically, which probably means that a number of relatively benign fractures are treated surgically. Accordingly, in regions with a small proportion of surgical treatment, the surgical complexity of fractures treated operatively ought to be higher. It is unknown which surgical method that is preferred in more complex fractures. Our clinical experience suggests that plating is more common than external fixation or pinning alone in complex cases. To avoid bias caused by differences in case mix in our analysis we have not analyzed records of patients treated with a combination of methods, e g plating and external fixation (presented in our study as “other”). Our study design does not, however, allow control for fracture pattern in all aspects, which we admit is a shortcoming of our study.

The regional differences we found regarding radial fracture treatment may also be explained by technology diffusion and financial incentives. The regions in Sweden that our study showed had the highest incidence rates of surgery, and use of internal fixation, were all situated in the densely populated southern parts of Sweden. Efficient marketing measures may influence the choice of treatment, especially when scientific support for its use is scarce. To minimize unmotivated variation for preference-sensitive conditions, especially in a perspective of delicate financial interests, dissemination of shared decision making tools is a high priority [32].

A factor that in part explains the allowance of differences in treatment recommendations for radial fractures is that indication for surgery of distal radius fractures is always relative. There may be a difference in hand and wrist function with or without surgery [2], but there is never a life-threatening condition based solely on a distal radius fracture. In contrast, for example in the case of hip fractures, the indication for surgery is more or less absolute, since mortality rates are high if surgical treatment is not undertaken. This fact explains a low regional variation for treatment of hip fractures [32].

Regional variation of treatment preferences has also been shown for other orthopaedic traumatic conditions, such as ankle fractures, as presented in a database study of Koval et al in the US [36]. Furthermore, a similar regional variation has been suggested for proximal humeral fracture treatment with a twenty- to thirtyfold difference between regions regarding proportions of operative treatment [37]. In other aspects of fracture care, unmotivated regional variation has been reported from Canada regarding hip fracture patients [38].

The strength of this study is its size, with a large unselected population of distal radius fracture patients in a well-defined time period. There are several limitations. There is no information on the severity of fractures or mechanism of injuries, nor on co-morbidities. However, this is the case for all regions. Neither is it known whether internal fixation means volar or dorsal plating (or both). There is no data on the specialty of the surgeon and our study does not present data for non-surgically treated patients.

We conclude that we found large regional variation regarding choice of treatment methods for distal radius fractures among different parts of Sweden during the period 2010–2013. To minimize medically unmotivated regional differences, decision-making regarding radius fracture treatment should involve best available scientific evidence, health economic considerations and patient preference involvement.
